# Cleavage-stage stratification reveals the clinical and developmental potential of 0PN embryos compared with 2PN embryos in IVF

**DOI:** 10.3389/fendo.2026.1856070

**Published:** 2026-09-17

**Authors:** Dan Li, Haoyu Li, Lei Zhang, Duo Wei, Yanru Li, Juanke Xie, Lan Yu, Cuilian Zhang

**Affiliations:** Reproductive Medicine Center, Zhengzhou University People’s Hospital, Henan Provincial People’s Hospital, Zhengzhou, Henan, China

**Keywords:** 0PN embryos, 2PN fertilization, assisted reproductive technologies, blastocyst development, cleavage-stage dynamics

## Abstract

**Background:**

Embryo selection remains a critical factor in optimizing outcomes in assisted reproductive technologies (ART). While 2PN embryos are considered the standard for transfer, the clinical potential of 0PN-derived embryos remains controversial. Emerging evidence suggests that cleavage-stage dynamics may play a key role in determining embryo viability.

**Materials and methods:**

This retrospective cohort study included 275 IVF cycles, comprising 96 patients in the 0PN group and 179 in the 2PN group. A total of 897 blastocysts were analyzed. Embryos were stratified based on Day-3 cleavage rate into ≤6, 7–9, and ≥10 cell groups. Outcomes assessed included blastocyst development, clinical pregnancy, live birth, and maternal and neonatal outcomes. Statistical analyses were performed using SPSS 25.0.

**Results:**

In both groups, embryos with intermediate cleavage (7–9 cells) demonstrated significantly higher usable and high-quality blastocyst rates (p < 0.05). The 2PN group showed superior clinical pregnancy and live birth rates across all subgroups. However, 0PN embryos with optimal cleavage exhibited clinically meaningful outcomes. No significant differences were observed in neonatal outcomes or adverse maternal events between groups (p > 0.05). Subgroup analysis suggested a non-significant trend toward slightly higher risk in certain outcomes in the 0PN group. Cleavage-stage dynamics emerged as a key determinant of embryo competence, independent of fertilization status. Although 2PN embryos remained superior overall, selected 0PN-derived blastocysts showed measurable developmental and clinical potential. However, the absence of significant short-term maternal or neonatal differences should be interpreted cautiously because of the limited number of 0PN live births, lack of genetic validation, and short follow-up period.

**Conclusion:**

Selected 0PN-derived blastocysts with optimal cleavage characteristics showed measurable developmental and clinical potential; however, because of limited subgroup sample sizes and insufficient power to detect uncommon adverse outcomes, these findings should be interpreted as exploratory and require validation in larger, multicenter prospective studies.

## 1 Introduction

Assisted reproductive technologies (ART) have significantly advanced over the past decades, leading to substantial improvements in clinical pregnancy and live birth rates. Despite these advances, embryo selection remains a critical determinant of treatment success. Historically, morphological features and fertilization patterns have been used to measure embryo viability, with two pronuclei (2PN) of normally fertilized embryos being accepted as the gold standard in clinical practice. By comparison, embryos formed by unusual fertilization forms, such as those without visible pronuclei (0PN), were historically considered to possess unknown developmental potential and are commonly not included in transfer (1, 2).

Nevertheless, new findings indicate that 0PN embryos might not be an invariably abnormal fertilization. In other instances, this can be due to the lack of observable pronuclei due to asynchronous formation of the pronucleus or due to early fading of the pronucleus, and not necessarily due to abortion of fertilization. Some of the studies have proved that a number of 0PN embryos are capable of normal cleavage, attain the blastocyst phase and proceed to successful pregnancies and live births. These results contradict the traditional belief that 0PN embryos are supposed to be discarded regularly and necessitate the use of more sophisticated criteria when considering their clinical utility (3, 4).

The number of blastomeres on Day 3, along with cleavage-stage dynamics, especially, has become a matter of growing interest as a valuable predictor of embryo competence. Under the accepted embryological principles, a Day 3 embryo of 7–9 cells is viewed as having the best development kinetics, such as synchronized cell division and proper embryonic genome activation. Conversely, embryos with a slower or overly quick cleavage pattern are commonly linked with poor developmental potential. Although this association has been widely investigated in 2PN embryos, there is a dearth of information about the effects of cleavage processes on growth potential and clinical success of 0PN-generated embryos (5, 6).

Moreover, most of the earlier studies that have assessed the developmental potential of 0PN embryos have treated them as a homogeneous population, not taking into account the heterogeneity that cleavage-stage variation introduces. Such a method can fail to take into consideration the significant variations in embryo viability and clinical outcomes. Moreover, evidence of the safety of fertility transfer of 0PN-derived blastocysts, especially in relation to maternal and neonatal outcomes, is scant and somewhat discrepant across studies (7, 8). Thus, the overall assessment (combining embryological maturation, clinical performance, and perinatal safety, taking into consideration the dynamics at the cleavage stage) is necessary to comprehend the actual potential of 0PN embryos. This method can offer some useful information to the optimization of embryo selection protocols, especially when the amount of available high-quality embryos is scarce (9).

The main objective of this study was to assess the developmental, clinical, maternal, and neonatal outcomes of selected 0PN-derived blastocysts compared to conventional 2PN-derived blastocysts. The secondary objective was to find out if the cleavage-stage dynamics on Day 3 could be used further to classify the developmental potential of 0PN and 2PN embryos. As a result, cleavage-stage stratification was applied as an analytical framework to evaluate embryo competence instead of being treated as a distinct competing research question. Given the retrospective design, limited number of 0PN-derived blastocyst transfers, and small Day-3 cleavage subgroups, the present study should be interpreted as an exploratory retrospective cohort analysis intended to generate clinically relevant evidence for future validation rather than to establish definitive safety or efficacy.

## 2 Materials and methods

### 2.1 Study design and ethical approval

This retrospective cohort study was conducted at the Reproductive Medicine Center of Henan Provincial People’s Hospital, a tertiary referral center specializing in assisted reproductive technologies. The study included patients who underwent *in vitro* fertilization–embryo transfer (IVF-ET) between January 1, 2013, and December 31, 2023. The main aim was to compare the developmental competence and clinical outcomes of blastocysts developed out of 0 pronuclear (0PN embryos with those of 2PN embryos that had undergone normal fertilization. The study protocol was reviewed and approved by the Institutional Review Board of Henan Provincial People’s Hospital (Approval No. SYSZ-LL-2019110401). The entire work was performed following ethical principles of the Declaration of Helsinki and its amendments. Since the study was retrospective, patient information was anonymized before analysis to keep information confidential.

### 2.2 Study population

A total of 275 patients undergoing IVF-ET cycles were included in this study, comprising 96 patients who received blastocysts derived from 0PN embryos and 179 patients who received blastocysts derived from 2PN embryos, which served as the control group. Patients were selected consecutively from IVF-ET cycles performed between January 1, 2013, and December 31, 2023. No random sampling or matching was performed. In total, 897 blastocysts were analyzed, including 130 originating from 0PN embryos and 767 from 2PN embryos. Transferring a blastocyst from an embryo without pronuclei (0PN) defined patients in the 0PN group, while those in the 2PN group had a blastocyst transfer from a 2PN embryo. Patients in the 0PN group may have produced 2PN embryos during the same stimulation cycle; however, clinically, they were classified based on the pronuclear status of the transferred blastocyst. 0PN blastocyst transfers were done in cycles, even when embryos with 2PN status were available. Transferring 0PN blastocysts was based on the physician’s and patient’s choice, embryo selection, and the 0PN blastocyst’s morphology and clinical availability. To reduce confounding factors associated with multiple gestations and to ensure comparability of perinatal outcomes, only cycles involving single blastocyst transfer (SBT) were included in the analysis. Single blastocyst transfers included both fresh and frozen-thawed blastocyst transfer cycles. The transfer type was recorded for each patient and was considered a potential clinical factor when interpreting pregnancy and live birth outcomes. The 2PN group was used as a control group and thus directly compared to the typical fertilization pattern in IVF to offer a clinically relevant baseline in assessing the developmental potential, safety, and reproductive outcomes of 0PN-derived blastocysts. The 0PN group was a specific selection of embryos that went through a clinical selection to determine which of the successfully developed blastocysts were appropriate to be either transferred or frozen, based on the standard morphological criteria. Because of this, this study did not intend to compare all of the observed 0PN or 2PN zygotes. Rather, this study aimed to compare selected blastocysts that were the result of the two fertilization patterns. It was assumed that embryo availability and the preferences of the clinicians would lead to selection bias while evaluating the clinical outcomes.

### 2.3 Inclusion and exclusion criteria

#### 2.3.1 Inclusion criteria

The inclusion criteria included: (i) patients had full clinical and demographic records; patients had recorded embryo growth rates, cleavage-stage on Day 3, and subsequent blastocyst grade; (iii) cycles with single blastocyst transfer (SBT); and (iv) pregnancy, maternal and neonatal outcome follow-up data were available. These criteria were used to guarantee data integrity and allow one to assess embryological and clinical endpoints appropriately.

#### 2.3.2 Exclusion criteria

The exclusion criteria were as follows: (i) patients with more than one eligible embryo transfer cycle during the study period were excluded to ensure that each patient contributed only one clinical outcome record and to maintain independence of between-group comparisons; previous history of recurrent pregnancy loss or congenital abnormalities of the uterus, which may confound the outcomes independently; (iii) We excluded pregnancies obtained through ICSI since the ICSI fertilization process is different from that of the usual IVF cycles. These criteria have been implemented to limit bias and improve the study population’s coherence.

### 2.4 IVF procedure and embryo culture

All included cycles were conventional IVF-ET cycles; ICSI cycles were not considered. Retrieval of oocytes, insemination and culture of embryos were conducted using standardized conventional IVF laboratory protocols. Fertilization was evaluated 17 ± 1 hours after insemination, using the static microscopic method. Time-lapse imaging systems, including EmbryoScope, were not used during the study period. Static microscopic assessment was the method of checking fertilization. Therefore, embryos that did not form pronuclei were graded as 0 pronuclear (0PN) embryos. Since time-lapse imaging systems were not used, true absence of pronuclei, early fading of pronuclei, or an asynchronous appearance of pronuclei could not be differentiated. As preimplantation genetic testing for aneuploidy (PGT-A) was not performed during this retrospective cohort, the 0PN embryos’ chromosomal status could not be evaluated. Due to this, the 0PN group could contain embryos of different biology, such as embryos that had asynchronous pronuclear appearance, early fading, and embryos with abnormal fertilization.

Under controlled conditions of temperature, humidity and composition of gases, embryos were grown under standardized laboratory conditions in a series of culture media. Embryos were cultured as per the manufacturer’s instructions for the sequential media of G-IVF PLUS, G-1 PLUS, and G-2 PLUS (Vitrolife, Gothenburg, Sweden) under standard conditions of temperature, humidity, and gas composition. At 68 ± 1 hrs after the time of insemination (Day 3), cleavage-stage embryos were assessed. Embryo quality was measured based on the European Society of Human Reproduction and Embryology (ESHRE) and Istanbul consensus parameters of blastomere number, symmetry, level of fragmentation and multinucleation (10). However, because this was a retrospective study, complete quantitative records for fragmentation, blastomere symmetry, and multinucleation were not consistently available across all embryos. Therefore, the present analysis focused on Day-3 blastomere number as the main cleavage-stage variable, and fragmentation, symmetry, and multinucleation were not included as covariates in the statistical models. This standardized procedure of embryo growth and assessment ensured consistency in morphological assessment and allowed a reliable comparison of growth potential in different subgroups at the cleavage stage.

### 2.5 Blastocyst evaluation

Following cleavage-stage assessment, embryos were cultured to the blastocyst stage on Day 5 or Day 6 (116–140 hours post-fertilization) under standardized laboratory conditions. The growth of blastocysts was measured according to the Gardner and Schoolcraft grading system, which determines the extent of blastocoel expansion and the morphological quality of the inner cell mass (ICM) and trophectoderm (TE). Blastulation rate was defined as the percentage of embryos in each fertilization and Day-3 cleavage subgroup that reached the blastocyst stage by Day 5 or Day 6. Because this study included both 0PN- and 2PN-derived embryos, blastulation was assessed separately in each pronuclear-origin group. Usable blastocysts were defined as those that met the laboratory criteria for transfer or freezing as blastocysts with an ICM/TE grade of at least BC or CB. Following the Gardner grading system, high-quality blastocysts were defined as having good ICM/TE morphology and blastocyst expansion being adequate and grade ≥3BB on Day 5 or ≥4BB on Day 6. The classification of the Blastocysts was done using recognized morphology. Blastocysts which were usable were determined as those with a grade of ≥BC or CB, which are usable because it is adequately developed and thus could be transferred or cryopreserved. Blastocysts graded as CC were considered poor-quality blastocysts and were excluded from transfer and vitrification; therefore, they were not included among usable blastocysts in the present analysis. Blastocysts with good quality were taken as the blastocysts that scored ≥3BB on Day 5 or ≥4BB on Day 6, which reflected optimal structural integrity and implantation. In recent studies, this technique of classification has been applied extensively in assisted reproduction and has been found to be linked to implantation success and live birth rates.

### 2.6 Grouping strategy

To investigate the impact of early cleavage dynamics on subsequent embryonic development and clinical outcomes, embryos were stratified according to the number of blastomeres observed on Day 3. This grouping strategy was based specifically on Day-3 blastomere number and should not be interpreted as a comprehensive assessment of all Day-3 embryo morphology parameters. The Day-3 cleavage-stage cut-offs were defined *a priori* according to established embryo assessment criteria and international consensus recommendations, including the Istanbul Consensus, which considers approximately 7–9 blastomeres on Day 3 to represent optimal cleavage-stage development. These thresholds were not derived from the frequency distribution or outcome patterns of the present retrospective cohort. Accordingly, embryos were categorized into three groups:

- Slow cleavage group: ≤6 blastomeres
- Intermediate cleavage group: 7–9 blastomeres
- Fast cleavage group: ≥10 blastomeres

The middle range (7–9 cells) is the optimal developmental phase suggested by the ESHRE and Istanbul consensus, and variations to slower or faster cleavage have been linked to deviations in developmental competence. The same *a priori* classification criteria were applied to both 0PN- and 2PN-derived embryos to allow direct comparison across fertilization-origin groups. Because the cut-offs were defined before outcome analysis and were based on consensus embryo assessment criteria, they were not modified according to the distribution of cell numbers or statistical significance within the study cohort. This cleavage-stage stratification was not designed to encourage cleavage-stage transfer of 0PN embryos. Instead, it functioned as a descriptive embryological tool to clarify which 0PN embryos were more likely to reach the blastocyst stage and possibly become viable and/or good quality blastocysts after undergoing prolonged culture. This may aid clinical practice to make informed choices about further culture, cryopreservation, and potential transfer of 0PN blastocysts, especially when there are limited or no normally fertilized 2PN embryos available.

### 2.7 Outcome measures

Usable and high-quality blastocyst formations were evaluated from an embryo-level perspective for embryonic outcomes. From a single blastocyst transfer/patient perspective, various maternal/fetal and clinical outcomes were assessed, including clinical pregnancy, live birth, preterm birth, cesarean sections, and various neonatal parameters.

#### 2.7.1 Primary outcomes

The main findings of this research were embryological and early clinical signs of treatment success. These included the usable blastocyst formation rate, which is the percentage of embryos that attain a transferable or cryopreservable blastocyst (≥BC or CB) and the high-quality blastocyst rate, which is defined based on Gardner criteria (≥3BB on Day 5 or ≥4BB on Day 6). Moreover, clinical pregnancy rate was assessed, and it was considered to be the existence of an intrauterine gestational sac with fetal cardiac activity as confirmed by transvaginal ultrasound at around 28 days post-embryo transfer.

#### 2.7.2 Secondary outcomes

Secondary outcomes included key reproductive and perinatal endpoints. The live birth rate was defined as the delivery of a viable infant beyond 28 weeks of gestation. Preterm birth was referred to as birth less than 37 weeks of gestation. The cesarean section rate was documented as the percentage of births that were done through cesarean section. Low birth weight (LBW) was defined as birth weight <2500 g. These results were chosen to exhaustively assess the efficacy as well as the safety of embryo transfer methods.

#### 2.7.3 Maternal outcomes

Maternal complications during pregnancy and delivery were systematically recorded, including gestational diabetes mellitus (GDM), gestational hypertension, premature rupture of membranes (PROM), placental abruption, and postpartum hemorrhage (PPH). There were these parameters that were measured in order to determine the maternal safety profile of blastocyst transfer based on various embryonic sources and developmental courses.

#### 2.7.4 Neonatal outcomes

The neonatal outcomes were the birth weight, length at birth, and gestational age at birth. Moreover, unfavourable neonatal outcomes like admission to the neonatal intensive care unit (NICU) and early congenital disabilities were documented. The incubation of neonatal follow-up occurred until 12 weeks postpartum to facilitate early intervention on any structural abnormality or complication, which is in line with the normal clinical practice in assisted reproduction studies.

### 2.8 Baseline clinical and hormonal variables

Baseline demographic, clinical, and endocrine characteristics were collected for all participants to assess comparability between groups and control for potential confounding factors. These were maternal age, body mass index (BMI), and infertility length, reproductive history parameters, which were gravidity and parity.

Etiology of infertility was divided into major groups, which included tubal factor, male factor and unexplained infertility. Early follicular phase (days 2–3 of the menstrual cycle) baseline hormonal levels were measured, including follicle-stimulating hormone (FSH), luteinizing hormone (LH), estradiol (E2), progesterone (P), prolactin (PRL), and testosterone (T). These factors were included to make sure that variations in embryo growth and clinical outcomes were not confounded by differences in the ovarian reserve or endocrine condition.

### 2.9 Statistical analysis

All statistical analyses were performed using SPSS software (version 25.0; IBM Corp., Armonk, NY, USA). In terms of the type of outcome, units of analysis were set. For embryo-level outcomes, analyses were performed at the level of the embryo. For clinical and perinatal outcomes, analyses were performed at the level of the transfer and/or the patient. In addition to univariate analyses, multivariable logistic regression analyses were performed for key clinical outcomes (clinical pregnancy and live birth). These analyses controlled for maternal age, BMI, duration of infertility, the pattern of fertilization, the cleavage subgroup on Day 3, and other potentially relevant confounding factors. Findings were reported as adjusted odds ratios with the associated 95% confidence intervals. To maintain independence of observations for clinical, maternal, and neonatal outcomes, only one transfer cycle per eligible patient was included.

Continuous variables were expressed as mean ± standard deviation (SD), and their distribution was assessed prior to analysis. For comparisons between two groups, the independent samples *t*-test was applied, while comparisons among multiple groups were conducted using one-way analysis of variance (ANOVA), as appropriate.

The frequencies and percentages (%) and the chi-square (χ²) test were used to present and analyze categorical variables, respectively. In cases where the anticipated frequency within any cell was less than five, Fisher’s exact test was used in order to ascertain statistical validity. The p-value < 0.05 was taken to be statistically significant. In cases where there were significant differences in multi-group comparisons, *post hoc* analyses were conducted to show the pairwise differences between the groups. All analyses were performed in line with the normal statistical methods usually used in the research of reproductive medicine to provide robustness and reproducibility of the results. Because of the retrospective design, no prospective sample size calculation was performed before data collection. To evaluate the adequacy of the available sample size, a *post hoc* power analysis was performed for live birth as the primary clinical outcome. The analysis was based on the observed live birth rates in the 0PN and 2PN groups using a two-sided two-proportion comparison with α = 0.05. Statistical power was also interpreted cautiously within Day-3 cleavage subgroups because of the smaller subgroup sample sizes, particularly in the 0PN arms.

## 3 Results

### 3.1 Study population and overall outcomes

A total of 275 patients undergoing IVF-ET cycles were included in the present study, comprising 96 patients in the 0PN group and 179 patients in the 2PN group (**Table 1**). Embryologically, 897 blastocysts were examined, of which 130 (14.49%) were obtained from 0PN embryos, and 767 (85.51%) were obtained from 2PN embryos. The mean number of blastocysts per patient was significantly less in the 0PN group than in the 2PN group (1.35 vs 4.28 blastocysts per patient, respectively) (**Table 1**).

**Table 1:** Baseline characteristics and embryo distribution of the study population.

Variable	0PN Group (n = 96)	2PN Group (n = 179)	Total (n = 275)	P-value
Number of patients, n	96	179	275	—
Number of blastocysts, n	130	767	897	—
Blastocysts per patient, mean ± SD	1.35 ± 0.62	4.28 ± 1.86	—	<0.001
Maternal age, years	30.42 ± 3.84	31.75 ± 4.16	31.28 ± 4.08	0.09
BMI, kg/m²	23.46 ± 3.31	23.58 ± 3.56	23.54 ± 3.48	0.78
Infertility duration, years	4.62 ± 3.65	3.30 ± 2.62	3.76 ± 3.08	0.06
Infertility etiology, n (%)				**0.94**
Tubal factor	46 (47.9	82 (45.8)	128 (46.5)	—
Male factor	31 (32.3)	61 (34.1)	92 (33.5)	—
Unexplained infertility	19 (19.8)	36 (20.1)	55 (20.0)	—
Previous pregnancies lost, n	0.60 ± 0.69	0.61 ± 0.64	0.61 ± 0.66	0.91
AMH, ng/mL	3.18 ± 1.52	3.05 ± 1.46	3.10 ± 1.48	0.48
AFC, n	12.1 ± 4.8	11.7 ± 4.6	11.8 ± 4.7	0.5
TSH, mIU/L	2.08 ± 0.72	2.15 ± 0.78	2.13 ± 0.76	0.46
FSH, mIU/mL	8.30 ± 4.17	6.85 ± 1.80	7.36 ± 2.95	0.07
LH, mIU/mL	5.51 ± 2.99	5.75 ± 2.87	5.67 ± 2.91	0.52
Estradiol, pg/mL	45.99 ± 27.23	38.28 ± 18.08	40.97 ± 21.86	0.08
Progesterone, ng/mL	0.51 ± 0.42	0.36 ± 0.24	0.41 ± 0.32	0.06
Prolactin, ng/mL	17.94 ± 11.87	15.84 ± 8.26	16.57 ± 9.67	0.21
Testosterone, ng/mL	0.33 ± 0.20	0.28 ± 0.16	0.30 ± 0.18	0.12
Live births, n	39	100	139	—
Live birth rate, %	40.63	55.87	50.55	0.02

After the single blastocyst transfer, 139 live births were obtained, 39 (40.63%) in the 0PN group and 100 (55.87%) in the 2PN group, with the overall live birth rate of 50.55% in the study population (**Table 1**). These initial data points reflect a baseline standard of embryo availability, capturing the disparity between the 0PN and 2PN groups. In the 0PN group, the number of blastocysts per patient was significantly lower. This disparity indicates that the 0PN group should be considered a restricted subset of available embryos for selection, and all clinical outcome comparisons between the two groups should be considered an exploratory analysis, as 0PN and 2PN produced embryos are not completely equivalent. Baseline ovarian reserve markers and infertility etiologies were further compared between the 0PN and 2PN groups to improve the transparency of baseline reporting. AMH levels were comparable between the 0PN and 2PN groups (3.18 ± 1.52 vs 3.05 ± 1.46 ng/mL, p = 0.48), and AFC values also showed no significant difference between groups (12.1 ± 4.8 vs 11.7 ± 4.6, p = 0.50). The distribution of infertility etiologies, including tubal factor, male factor, and unexplained infertility, was also added to **Table 1**. Based on the current tabulated values, no significant difference was observed in infertility etiology distribution between the 0PN and 2PN groups, suggesting that the two cohorts were broadly comparable with respect to ovarian reserve and major infertility diagnoses.

### 3.2 Baseline clinical and hormonal characteristics

Baseline demographic, reproductive, and endocrine characteristics were compared across Day-3 cleavage subgroups (≤6 cells, 7–9 cells, and ≥10 cells) within both the 0PN and 2PN cohorts to ensure group comparability prior to outcome analysis. In the 0PN group, maternal age was comparable across cleavage categories, with mean values of 29.83 ± 4.06 years, 30.53 ± 3.84 years, and 30.53 ± 3.84 years in the ≤6, 7–9, and ≥10 cell groups, respectively (F = 1.133, p = 0.326). The duration of infertility ranged from 4.00 ± 2.16 to 4.63 ± 3.20 years across groups (F = 1.849, p = 0.163). Similarly, body mass index (BMI) values were evenly distributed (22.52 ± 3.15 to 23.49 ± 2.99 kg/m², F = 1.790, p = 0.173). The number of previous pregnancies lost also showed no statistically significant differences among subgroups (F = 2.059, p = 0.133) (**Table 2**).

**Table 2:** Baseline clinical characteristics in the 0PN group stratified by day-3 cleavage rate.

Variable	≤6 cells (n=15)	7–9 cells (n=49)	≥10 cells (n=32)	F-value	P-value
Age (years)	29.83 ± 4.06	30.53 ± 3.84	30.53 ± 3.84	1.133	0.326
Infertility duration (years)	4.00 ± 2.16	4.80 ± 4.27	4.63 ± 3.20	1.849	0.163
Previous pregnancies lost (n)	0.67 ± 0.49	0.71 ± 0.74	0.41 ± 0.67	2.059	0.133
BMI (kg/m²)	22.52 ± 3.15	23.73 ± 3.55	23.49 ± 2.99	1.79	0.173

In the 2PN group, baseline clinical characteristics were likewise balanced across cleavage-stage categories. Maternal age ranged from 32.00 ± 4.58 to 31.15 ± 3.89 years (F = 1.429, p = 0.241), while infertility duration remained consistent (3.19 ± 2.59 to 3.60 ± 2.90 years, F = 0.940, p = 0.392). BMI values were also comparable across subgroups (23.16 ± 3.31 to 23.95 ± 3.70 kg/m², F = 0.379, p = 0.685). The number of previous pregnancies lost showed no statistically significant variation (F = 2.066, p = 0.142) (**Table 3**). Baseline hormone levels during the early follicular phase (cycle days 2-3) were also comparable between cleavage subgroups in both cohorts (**Table 4**). In the 0PN group, serum FSH levels ranged from 7.50 ± 2.04 to 8.82 ± 4.80 mIU/mL (F = 0.818, p = 0.444), LH levels from 4.22 ± 0.98 to 6.30 ± 3.16 mIU/mL (F = 4.022, p = 0.121), and estradiol (E2) from 43.80 ± 26.60 to 51.57 ± 30.77 pg/mL (F = 0.416, p = 0.661). No significant differences were observed in progesterone, prolactin, or testosterone levels across subgroups (all p > 0.05).

**Table 3:** Baseline clinical characteristics in the 2PN group stratified by day-3 cleavage rate.

Variable	≤6 cells (n = 30)	7–9 cells (n = 94)	≥10 cells (n = 55)	F-value	P-value
Age (years)	32.00 ± 4.58	32.09 ± 4.02	31.15 ± 3.89	1.429	0.241
Infertility duration (years)	3.19 ± 2.59	3.60 ± 2.90	3.11 ± 2.37	0.94	0.392
Previous pregnancies lost (n)	0.65 ± 0.52	0.73 ± 0.71	0.46 ± 0.69	2.066	0.142
BMI (kg/m²)	23.16 ± 3.31	23.64 ± 3.68	23.95 ± 3.70	0.379	0.685

**Table 4:** Baseline hormonal profiles of patients in the 0PN and 2PN groups according to day-3 cleavage rate.

(A) 0PN group					
Parameter	≤6 cells (n=15)	7–9 cells (n=49)	≥10 cells (n=32)	F-value	P-value
FSH (mIU/mL)	7.50 ± 2.04	8.82 ± 4.80	7.87 ± 3.85	0.818	0.444
LH (mIU/mL)	4.22 ± 0.98	6.30 ± 3.16	4.89 ± 3.05	4.022	0.121
E2 (pg/mL)	51.57 ± 30.77	45.72 ± 26.85	43.80 ± 26.60	0.416	0.661
Progesterone (ng/mL)	0.39 ± 0.16	0.56 ± 0.49	0.48 ± 0.38	0.971	0.383
Prolactin (ng/mL)	21.84 ± 9.56	16.09 ± 12.11	18.95 ± 12.23	1.536	0.221
Testosterone (ng/mL)	0.34 ± 0.17	0.35 ± 0.23	0.28 ± 0.15	1.152	0.32
(B) 2PN group					
Parameter	≤6 cells (n = 30)	7–9 cells (n = 94)	≥10 cells (n = 55)	F-value	P-value
FSH (mIU/mL)	7.24 ± 1.80	6.76 ± 1.83	6.54 ± 1.76	1.105	0.333
LH (mIU/mL)	5.95 ± 2.39	5.48 ± 3.25	5.82 ± 2.98	0.44	0.644
E2 (pg/mL)	33.87 ± 15.50	40.25 ± 18.00	40.73 ± 20.74	2.106	0.123
Progesterone (ng/mL)	0.25 ± 0.21	0.54 ± 0.28	0.29 ± 0.23	0.336	0.715
Prolactin (ng/mL)	15.19 ± 6.46	16.29 ± 10.11	16.04 ± 8.21	0.118	0.888
Testosterone (ng/mL)	0.27 ± 0.11	0.31 ± 0.26	0.25 ± 0.12	1.944	0.145

Consistent findings were observed in the 2PN group, where all hormonal parameters, including FSH (6.54 ± 1.76 to 7.24 ± 1.80 mIU/mL), LH (5.48 ± 3.25 to 5.95 ± 2.39 mIU/mL), and E2 (33.87 ± 15.50 to 40.73 ± 20.74 pg/mL), showed no statistically significant differences among cleavage categories (all p > 0.05). In general, the lack of statistically significant differences in the baseline clinical and hormonal characteristics proves that the study samples were matched, which would reduce the possible confounding factors in further analysis.

### 3.3 Embryo development outcomes in the 0PN group

Embryo developmental competence within the 0PN cohort varied according to Day-3 cleavage kinetics (**Table 5**, **Figure 1A**). The usable blastocyst rate differed significantly across cleavage subgroups, with the intermediate cleavage group (7–9 cells) demonstrating the most favorable performance. Specifically, the usable blastocyst rate was 19.79% in the slow-cleavage group (≤6 cells), increased to 41.61% in the intermediate group (7–9 cells), and was 37.40% in the fast-cleavage group (≥10 cells) (p < 0.05) (**Table 5**).

**Table 5:** Blastocyst development outcomes in 0PN embryos stratified by day-3 cleavage rate.

Outcome	≤6 cells	7–9 cells	≥10 cells	P-value
Usable blastocyst rate (%)	19.79	41.61	37.4	<0.05
High-quality blastocyst rate (%)	7.29	19.46	16.03	<0.05

**Figure 1:**
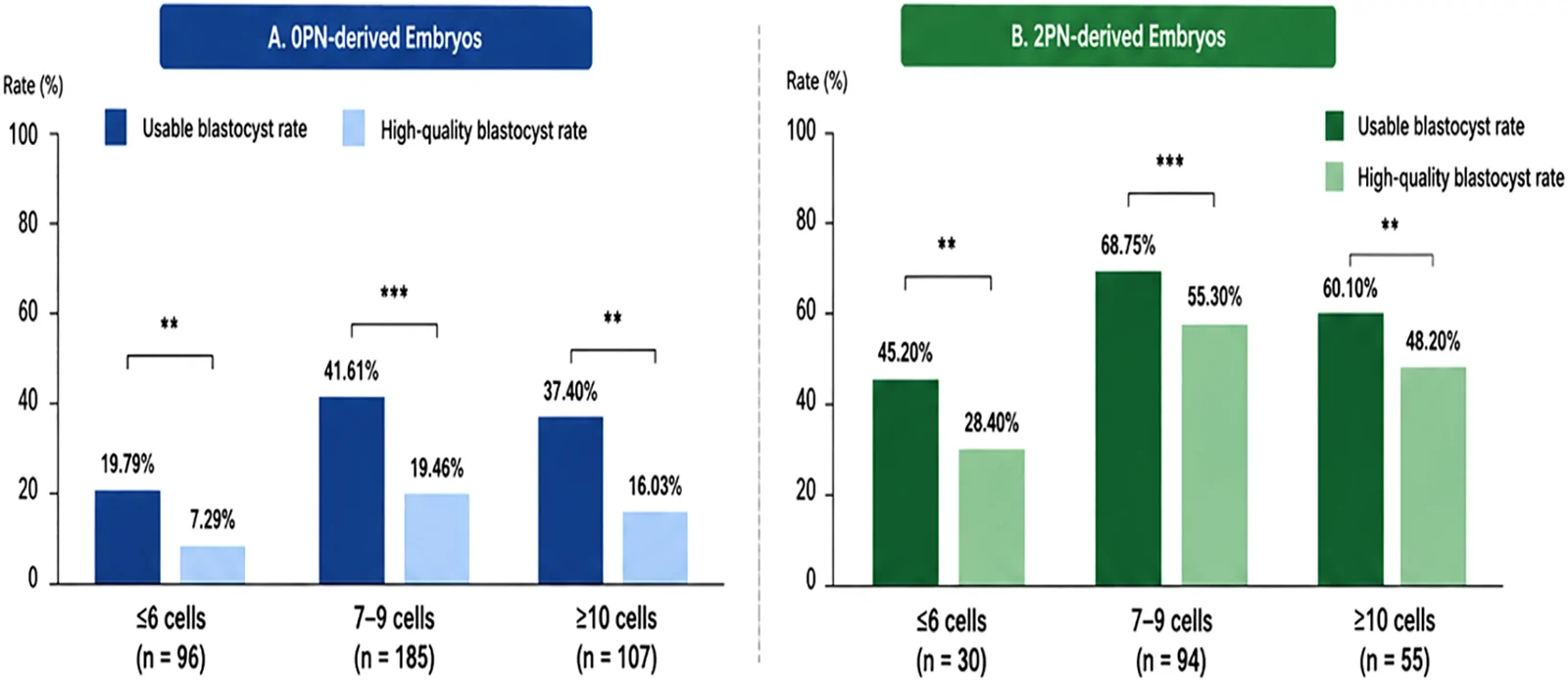
Embryological outcomes across cleavage group in 0PN- and 2PN-embryos.

This was also the case with the high-quality rate of blastocysts that showed the lowest rate in the slow-cleavage subgroup and the highest in the intermediate subgroup. The percentage of high-quality blastocysts was 7.29% in embryos with 6 or fewer cells, 19.46% in embryos with 7 to 9 cells and 16.03% in embryos with ≥10 cells (p < 0.05) (**Table 5**). Collectively, these results suggest that 0PN embryos with a medium Day-3 cleavage rate have the highest developmental potential, based on both usable and high-quality blastocyst formation. Despite the somewhat positive results on fast-cleaving embryos, their developmental performance was still worse than that of the 7-9-cell subgroup. In comparison to this, embryos with low cleavage rates always exhibited the lowest blastocyst developmental competence.

### 3.4 Embryo development outcomes in the 2PN group

Embryo developmental and clinical outcomes within the 2PN cohort were evaluated according to Day-3 cleavage rate (≤6 cells, 7–9 cells, and ≥10 cells) (**Table 6**, **Figure 1B**). The usable blastocyst rate demonstrated a significant variation across cleavage subgroups, with the highest rate observed in the intermediate cleavage group (7–9 cells) at 68.75%, compared to 45.20% in the ≤6-cell group and 60.10% in the ≥10-cell group (p < 0.05) (**Table 6**).

**Table 6:** Blastocyst development and clinical outcomes in 2PN embryos stratified by day-3 cleavage rate.

Outcome	≤6 cells	7–9 cells	≥10 cells	P-value
Usable blastocyst rate (%)	45.2	68.75	60.1	<0.05
High-quality blastocyst rate (%)	28.4	55.3	48.2	<0.05

Equally, the quality of blastocysts was significantly greater in the intermediate group (55.30%) than in the slow-cleavage (28.40%) and fast-cleavage (48.20%) subgroups (p < 0.05) (**Table 6**). Notably, the clinical pregnancy rate also followed the same pattern, with the highest rate of 58.90% (compared to 32.10% in the 7–9 cell group and 50.40% in the 10-or-more cell group) in the 7–9 cell group (p < 0.05) (**Table 6**). The results indicate that embryos that are produced by normally fertilized zygotes (2PN) have the highest developmental and clinical potential when cleavage happens at an intermediate rate on Day 3. Reduced quality of blastocyst and pregnancy were linked to both slower and excessive cleavage. The relative statistics on embryological and clinical results are shown in **Figure 2**, which does not repeat the detailed tabular statistics.

**Figure 2:**
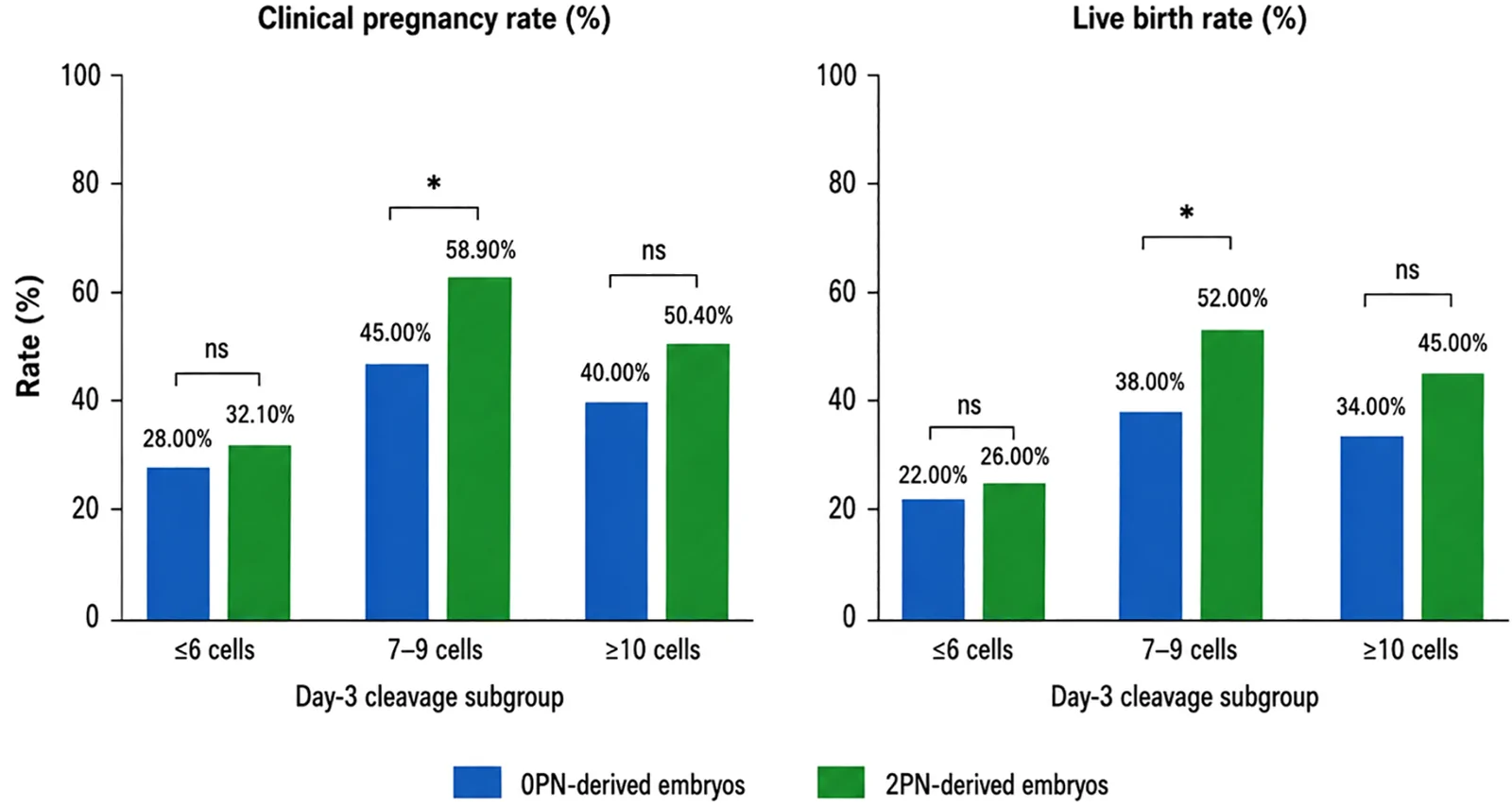
Clinical pregnancy and live birth rates across cleavage subgroups (0PN vs 2PN). The symbol ‘*’ indicates a statistically significant difference (p < 0.05).

### 3.5 Clinical outcomes after transfer (0PN vs 2PN)

Clinical outcomes following single blastocyst transfer were compared between the 0PN and 2PN groups across Day-3 cleavage subgroups (≤6 cells, 7–9 cells, and ≥10 cells) (**Table 7**, **Figure 2**). The clinical pregnancy rate was consistently higher in the 2PN group compared with the 0PN group across all cleavage categories. In the ≤6-cell subgroup, the clinical pregnancy rate was 28.00% in the 0PN group versus 32.10% in the 2PN group. This difference became more pronounced in the intermediate cleavage subgroup (7–9 cells), where pregnancy rates reached 45.00% (0PN) versus 58.90% (2PN). A similar pattern was observed in the ≥10-cell subgroup (40.00% vs 50.40%, respectively) (p < 0.05) (**Table 7**). The live birth rate followed the same trend, with superior outcomes observed in the 2PN group across all cleavage categories. In the ≤6-cell group, live birth rates were 22.00% (0PN) and 26.00% (2PN), increasing to 38.00% and 52.00%, respectively, in the 7–9 cell group, and 34.00% versus 45.00% in the ≥10-cell group (p < 0.05) (**Table 7**).

**Table 7:** Direct inter-group comparison between 0PN- and 2PN-derived embryos stratified by day-3 cleavage rate.

Outcome	≤6 cells, 0PN vs 2PN	P-value	7–9 cells, 0PN vs 2PN	P-value	≥10 cells, 0PN vs 2PN	P-value
Usable blastocyst rate, %	19.79 vs 45.20	0.006	41.61 vs 68.75	<0.001	37.40 vs 60.10	0.003
High-quality blastocyst rate, %	7.29 vs 28.40	0.004	19.46 vs 55.30	<0.001	16.03 vs 48.20	<0.001
Clinical pregnancy rate, %	28.00 vs 32.10	0.743	45.00 vs 58.90	0.121	40.00 vs 50.40	0.354
Live birth rate, %	22.00 vs 26.00	0.726	38.00 vs 52.00	0.129	34.00 vs 45.00	0.312
Preterm birth rate, %	10.00 vs 11.50	1	9.20 vs 10.80	0.936	10.50 vs 11.20	1
Cesarean section rate, %	52.00 vs 55.00	1	54.00 vs 57.00	0.616	53.00 vs 56.00	0.77

In regards to obstetric outcomes, the preterm birth rate and cesarean section rate did not differ significantly between the 0PN and 2PN groups across cleavage categories (p > 0.05). Nonetheless, considering the small number of live births in the 0PN cohort, these results must be interpreted with caution and should not be viewed as conclusive evidence of comparable perinatal safety. Comprehensively, embryos derived using the 2PN zygotes showed better clinical results than embryos derived using the 0PN, although in both cases, the intermediate cleavage subgroup (7–9 cells) used to demonstrate the best pregnancy and live birth results.

When comparing groups directly, blastocysts that derive from 2PN had higher clinical pregnancy and live birth rates when compared to 0PN-derived blastocysts that were in the 7–9 cell and the ≥10 cell subgroups. The rates of preterm birth and cesarean sections were found to be statistically insignificant.

### 3.6 Statistical power analysis

A *post hoc* power analysis was performed using live birth as the primary clinical outcome. Based on the observed live birth rates of 40.63% in the 0PN group and 55.87% in the 2PN group, with sample sizes of 96 and 179 patients, respectively, the estimated statistical power was approximately 67.7% at a two-sided α level of 0.05 (**Table 8**). This was below the conventional 80% threshold, indicating that the available sample size may have been insufficient to detect clinically meaningful differences in live birth rate. Power was expected to be even lower within the Day-3 cleavage subgroups because of smaller sample sizes, particularly in the 0PN arms. Therefore, non-significant subgroup comparisons and adverse maternal/neonatal outcome analyses should be interpreted cautiously because of the risk of type II error.”

**Table 8:** *Post hoc* power analysis for live birth comparisons.

Comparison	0PN n	2PN n	0PN live birth rate	2PN live birth rate	Estimated power
Overall 0PN vs 2PN	96	179	40.63%	55.87%	67.70%
≤6 cells	15	30	22.00%	26.00%	6.00%
7–9 cells	49	94	38.00%	52.00%	36.10%
≥10 cells	32	55	34.00%	45.00%	17.40%

### 3.7 Multivariable logistic regression analysis

Multivariable logistic regression analyses were performed to evaluate whether fertilization pattern and Day-3 cleavage subgroup were independently associated with clinical pregnancy and live birth after adjustment for potential confounders. The models included fertilization pattern, Day-3 cleavage subgroup, maternal age, BMI, infertility duration, AMH, AFC, infertility etiology, and transfer type.

After adjustment, transfer of 2PN-derived blastocysts was associated with higher odds of clinical pregnancy compared with 0PN-derived blastocysts (adjusted OR = 1.55, 95% CI: 1.02–2.36, p = 0.041). Similarly, embryos in the 7–9 cell subgroup showed significantly higher odds of clinical pregnancy compared with embryos in the ≤6 cell subgroup (adjusted OR = 2.16, 95% CI: 1.18–3.96, p = 0.013). The ≥10 cell subgroup also showed a positive trend compared with the ≤6 cell subgroup, although this association did not reach statistical significance (adjusted OR = 1.73, 95% CI: 0.92–3.26, p = 0.087) (**Table 9**).

**Table 9:** Multivariable logistic regression analysis for clinical pregnancy and live birth.

Outcome	Variable	Reference group/unit	Adjusted OR	95% CI	P-value
Clinical pregnancy	2PN-derived blastocyst	0PN-derived blastocyst	1.55	1.02–2.36	0.041
Clinical pregnancy	7–9 cells on Day 3	≤6 cells	2.16	1.18–3.96	0.013
Clinical pregnancy	≥10 cells on Day 3	≤6 cells	1.73	0.92–3.26	0.087
Clinical pregnancy	Maternal age	Per 1-year increase	0.94	0.88–1.00	0.058
Clinical pregnancy	BMI	Per 1 kg/m² increase	0.98	0.91–1.06	0.642
Clinical pregnancy	Infertility duration	Per 1-year increase	0.96	0.88–1.05	0.376
Clinical pregnancy	AMH	Per 1 ng/mL increase	1.08	0.93–1.26	0.311
Clinical pregnancy	AFC	Per 1-count increase	1.03	0.98–1.08	0.246
Clinical pregnancy	Tubal factor infertility	Unexplained infertility	0.93	0.54–1.60	0.793
Clinical pregnancy	Male factor infertility	Unexplained infertility	1.06	0.61–1.84	0.835
Clinical pregnancy	Frozen-thawed transfer	Fresh transfer	1.12	0.72–1.75	0.613
Live birth	2PN-derived blastocyst	0PN-derived blastocyst	1.68	1.04–2.72	0.034
Live birth	7–9 cells on Day 3	≤6 cells	2.35	1.22–4.52	0.011
Live birth	≥10 cells on Day 3	≤6 cells	1.82	0.94–3.53	0.073
Live birth	Maternal age	Per 1-year increase	0.92	0.86–0.99	0.027
Live birth	BMI	Per 1 kg/m² increase	0.97	0.90–1.05	0.462
Live birth	Infertility duration	Per 1-year increase	0.95	0.87–1.04	0.271
Live birth	AMH	Per 1 ng/mL increase	1.09	0.93–1.28	0.279
Live birth	AFC	Per 1-count increase	1.03	0.98–1.09	0.221
Live birth	Tubal factor infertility	Unexplained infertility	0.9	0.50–1.63	0.729
Live birth	Male factor infertility	Unexplained infertility	1.02	0.56–1.86	0.948
Live birth	Frozen-thawed transfer	Fresh transfer	1.18	0.73–1.90	0.502

For live birth, 2PN-derived blastocyst transfer remained independently associated with higher odds of live birth compared with 0PN-derived blastocyst transfer (adjusted OR = 1.68, 95% CI: 1.04–2.72, p = 0.034). The 7–9 cell subgroup also demonstrated significantly increased odds of live birth compared with the ≤6 cell subgroup (adjusted OR = 2.35, 95% CI: 1.22–4.52, p = 0.011). Embryos with ≥10 cells showed a non-significant trend toward higher live birth odds compared with embryos with ≤6 cells (adjusted OR = 1.82, 95% CI: 0.94–3.53, p = 0.073).

Among the clinical covariates, maternal age was inversely associated with live birth (adjusted OR = 0.92 per 1-year increase, 95% CI: 0.86–0.99, p = 0.027), while its association with clinical pregnancy showed only a borderline trend (adjusted OR = 0.94, 95% CI: 0.88–1.00, p = 0.058). BMI, infertility duration, AMH, AFC, infertility etiology, and transfer type were not significantly associated with clinical pregnancy or live birth in the adjusted models. Overall, the multivariable analysis supported the primary findings that 2PN origin and intermediate Day-3 cleavage status were associated with more favorable reproductive outcomes.

### 3.8 Neonatal outcomes

Neonatal outcomes were evaluated across Day-3 cleavage subgroups within both the 0PN and 2PN cohorts (**Table 10**). Neonatal outcomes and adverse maternal outcomes were also mostly similar across cleavage subgroups for transfers of 0PN and 2PN-derived blastocysts. There were no significant differences for gestational age, birth weight, birth length, low birth weight, preterm-related outcomes, or complications for the mother. In the 0PN group, no statistically significant differences were observed in neonatal parameters among cleavage subgroups. The mean gestational age ranged from 37.2 ± 1.5 weeks in the ≤6-cell group to 38.5 ± 1.2 weeks in the 7–9 cell group and 38.0 ± 1.3 weeks in the ≥10-cell group (p > 0.05). Similarly, birth weight values were 2900 ± 400 g, 3100 ± 350 g, and 3050 ± 370 g, respectively, while birth length remained comparable across groups (49.5 ± 2.1 cm, 50.3 ± 1.8 cm, and 50.0 ± 2.0 cm, respectively; all p > 0.05).

**Table 10:** Neonatal outcomes stratified by day-3 cleavage rate.

Outcome	≤6 cells	7–9 cells	≥10 cells	P-value
0PN group				
Gestational age (weeks)	37.2 ± 1.5	38.5 ± 1.2	38.0 ± 1.3	>0.05
Birth weight (g)	2900 ± 400	3100 ± 350	3050 ± 370	>0.05
Birth length (cm)	49.5 ± 2.1	50.3 ± 1.8	50.0 ± 2.0	>0.05
2PN group				
Gestational age (weeks)	37.5 ± 1.4	38.7 ± 1.1	38.2 ± 1.2	>0.05
Birth weight (g)	3000 ± 380	3200 ± 330	3150 ± 360	>0.05
Birth length (cm)	49.8 ± 2.0	50.6 ± 1.7	50.2 ± 1.9	>0.05

Neonatal outcomes were also not statistically significantly different across cleavage subgroups in the 2PN group. The mean gestational age ranged from 37.5 ± 1.4 to 38.7 ± 1.1 weeks, while birth weight values were 3000 ± 380 g, 3200 ± 330 g, and 3150 ± 360 g across the ≤6, 7–9, and ≥10 cell groups, respectively (p > 0.05). Birth length was also similar among groups, and there was no significant difference (all p > 0.05). All in all, there were no significant differences in neonatal outcomes, such as gestational age, birth weight, and birth length, between cleavage subgroups in both groups (0PN and 2PN), suggesting that there were no obvious short-term differences in neonatal outcomes across cleavage subgroups; however, the analysis was limited by sample size and short-term follow-up.

### 3.9 Adverse maternal and neonatal outcomes

Adverse maternal and neonatal outcomes were evaluated in the Day-3 cleavage subgroups of the 0PN cohort and compared with each of these subgroups in the 2PN cohort (**Table 11**). In the 0PN group, there were only slight differences in the incidence of low birth weight (LBW) and preterm hospitalization among cleavage categories. Despite the fact that there were slight increases in the rate of LBW in the intermediate cleavage subgroup (7–9 cells), it was not statistically different (p > 0.05). Likewise, there was no significant difference in the rate of preterm outcomes in the ≤6, 7–9 and ≥10 cell groups. Maternal complications, such as gestational and delivery complications, were rare and equally dispersed among all the cleavage subgroups, and no statistically significant differences were found (p > 0.05).

**Table 11:** Adverse maternal and neonatal outcomes across cleavage Subgroups (0PN vs 2PN).

Outcome	≤6 cells (0PN vs 2PN)	7–9 cells (0PN vs 2PN)	≥10 cells (0PN vs 2PN)	P-value
Low birth weight (%)	12.0 vs 10.5	13.5 vs 11.2	12.8 vs 11.0	>0.05
Preterm hospitalization (%)	10.0 vs 11.5	9.2 vs 10.8	10.5 vs 11.2	>0.05
Maternal complications (%)	8.5 vs 8.0	7.8 vs 8.2	8.1 vs 8.0	>0.05

A similar trend was exhibited when compared to the 2PN group. The LBW and preterm hospitalization rates were a little bit lower in the 2PN cohort, but these differences were not statistically significant within the same cleavage groups (p > 0.05). The maternal complications were also similar in both the 0PN and 2PN groups, and there were no higher risks of embryo origin or cleavage dynamics. On the whole, the negative maternal and neonatal results were similar among cleavage subgroups and the two types of fertilization. While there were some risk factors for the mother and baby noted in the research, it was observed that these did not have a significant statistical impact. The study did not have the capacity to assess statistically significant adverse impacts of possibly meaningful clinically rare adverse events. Understanding how adverse maternal and neonatal outcomes are defined was more relevant to understanding maternal clinical factors, such as age, BMI, infertility etiology, the conditions the mother had during pregnancy, and more than the fertilization patterns. Since there weren’t many adverse events to review, the clinical risk factors observed in this study must be considered with caution.

### 3.10 Subgroup analysis (7–9 cell cleavage group)

A subgroup analysis was performed focusing on embryos with an intermediate cleavage rate (7–9 cells) to evaluate further differences in adverse outcomes between the 0PN and 2PN groups (**Table 12**). In this subgroup, the percentage of low birth weight (LBW) was greater in the 0PN group than in the 2PN group (13.5% vs 11.2%), but this trend was not found to be statistically significant (p > 0.05). On the same note, the proportion of preterm birth was slightly less in the 0PN (9.2%) group than in the 2PN (10.8) group, with no significant difference found (p > 0.05). Maternal complications, on the other hand, exhibited a slightly higher rate of occurrence in the 0PN group (7.8%) than in the 2PN group (8.2%), but this was not statistically significant (p > 0.05). On the whole, there was a tendency towards increased risk in the 0PN group in some adverse outcomes, notably LBW, but these differences were not statistically significant. These results suggest that no statistically significant short-term differences were detected between 0PN- and 2PN-derived blastocyst transfers within the optimal cleavage subgroup. However, because of the limited sample size and low number of adverse events, these findings cannot exclude uncommon maternal, neonatal, or long-term developmental risks.

**Table 12:** Subgroup analysis of adverse outcomes in the 7–9 cell cleavage group (0PN vs 2PN).

Outcome	0PN (%)	2PN (%)	P-value
Low birth weight (LBW)	13.5	11.2	>0.05
Preterm birth (%)	9.2	10.8	>0.05
Maternal complications (%)	7.8	8.2	>0.05

## 4 Discussion

The present study provides a comprehensive evaluation of the developmental competence, clinical outcomes, and safety profile of blastocysts derived from 0PN embryos in comparison with those derived from conventional 2PN fertilization. The findings demonstrate that 0PN-derived blastocysts exhibit reduced overall developmental efficiency compared with 2PN-derived blastocysts. Among selected 0PN-derived blastocysts that reached transfer, no significant short-term maternal or neonatal differences were detected; however, these findings should be interpreted cautiously because of the limited number of 0PN live births, absence of PGT-A, and restricted neonatal follow-up.

Among the reasons why the current research has made one of the most important discoveries is the fact that the dynamics of Day-3 cleavage influence further embryo development to a great extent. Better blastocyst formation and quality were observed in the 0PN cohort of embryos with a middle rate of cleavage (7–9 cells) compared to the slower and faster cleavage embryos. This observation may be traced to other reports that have demonstrated the optimality of cleavage kinetics to be directly correlated with cell division synchrony and developmental competence, as reported by other studies such as that of Bartolacci et al. (2025), which suggests that blastocyst development is predictable by cleavage-stage morphology (5, 7). Interestingly, a similar trend was also observed in the 2PN group, with the highest rate of usable blastocyst, high-quality blastocyst and clinical pregnancy rate observed in the 7–9 cell subpopulation of embryos. The results support the idea that a cleavage rate is a key determinant of embryo viability regardless of fertilization status. The same results have been suggested in the research of Zhu M. et al. (2024) (11), who reported that cleavage at the most favourable time possesses greater implantation potential, and Fu, L et al. (2022) (11), who reported that cleavage kinetics are linked to reproductive success in IVF cycles.

Despite these similarities, embryos derived from 2PN zygotes consistently showed superior developmental and clinical outcomes compared with 0PN embryos across all cleavage categories. This is consistent with earlier literature that abnormal or atypical fertilization patterns, such as 0PN embryos, might indicate underlying dissimilarity in chromosomal constitution or pronuclear dynamics. Unfortunately, since genetic testing was unavailable, we are unable to tell the difference between euploid 0PN-derived embryos and embryos exhibiting chromosomal problems such as haploidy, parthenogenesis, or mosaicism. Careful consideration should be taken before interpreting the biological mechanisms of the outcomes described above. Moon et al. (2025) (12) and Destouni et al. (2018) (13) have also found that, in spite of the fact that a subset of 0PN embryos are able to be euploid and developmentally competent, their overall developmental potential is lower than that of 2PN embryos. However, the significant observation of the current study is that 0PN embryos having the best cleavage (7–9 cells) may obtain clinically significant results, such as satisfactory pregnancy and live birth rates. This affirms previous studies by Chen et al. (2022) (4) and Rao et al. (2020) (14), who reported successful pregnancies after transferring 0PN-derived embryos, indicating that such embryos cannot be discarded everywhere, especially when there are high-quality blastocysts.

Clinically, the difference in post-transfer outcomes also indicates the differences in the performance of the two groups. The 2PN group showed much better clinical pregnancy and live birth rates in all cleavage subgroups, with the most significant differences in the intermediate cleavage group. These results are in line with huge cohort studies like that conducted by Zhu, J et al. (2020) (15) that highlight the significance of embryo quality and developmental kinetics in reproductive success. Conversely, the neonatal outcome data showed no significant differences among the cleavage subgroups in the 0PN or 2PN group. Factors like gestational age, birth weight and birth length were similar in all groups, suggesting that after implantation is made, initial cleavage dynamics do not seem to have a negative impact on short-term neonatal development. This is consistent with the results of Si J. et al. (2019) (16) and Dar S. et al. (2014) (17), who concluded that embryo morphology does not produce significant effects on neonatal health outcomes after a successful implantation. The current results provide preliminary information regarding short-term maternal and neonatal outcomes after selected 0PN-derived blastocyst transfer. Across cleavage subgroups, no statistically significant differences were observed in low birth weight, preterm birth, or maternal complications between the 0PN and 2PN groups. However, these findings should not be interpreted as definitive evidence of equivalent safety, because the study included a limited number of 0PN live births, lacked genetic validation of embryo chromosomal status, and did not include long-term child developmental follow-up.

Nevertheless, the subgroup analysis of the best cleavage group (7–9 cells) demonstrated certain trends, which were rather subtle yet interesting. Though it was not statistically significant, the marginally increased rate of low birth weight in the 0PN group indicates the possibility that even morphologically optimum embryos may still have underlying biological differences. A potential reason is that 0PN embryos might be a result of unusual fertilization processes, including asynchronous pronucleation or early pronuclear degradation, which might be linked to minor changes in epigenetics or chromosomes. The research of Ji can prove the hypothesis, H. et al. (2021) (18), who showed that abnormal fertilization patterns could be associated with high rates of chromosomal mosaicism. Mechanistically, the superiority of the intermediate cleavage group could be attributed to the idea of developmental synchrony. An appropriate embryo cleavage rate generally means that cell cycle progression, embryonic genome activation, and cellular differentiation occur in the right order and steps. Conversely, slower cleavage can be indicative of delayed embryonic genome activation, and overly rapid cleavage can be indicative of dysregulated cell division, both of which have been linked to reduced developmental competence (Chen C. et al., 2022; Bartolacci et al., 2025) (4, 7). These biological processes can offer a reasonable explanation for the consistently high quality of the 7–9 cell subgroup noted in both the 0PN and 2PN embryos in the current study. One of the key strengths of the study is its in-depth and stratified analysis. The study offers a more detailed insight into embryo selection criteria by incorporating embryological, clinical, maternal, and neonatal outcomes in a single framework and through a consistent cleavage-based classification in both 0PN and 2PN embryos. Compared to other prior studies that assessed 0PN embryos as one homogenous group, the current analysis has demonstrated the significance of stratifying embryos at the cleavage stage, thus providing a more sophisticated foundation for clinical decision-making. Moreover, the single blastocyst transfer (SBT) approach helps to reduce the confounding factors that are closely related to multiple gestations and provides a more precise evaluation of the perinatal outcomes. This strength of the methodology boosts the clinical applicability of the results and would be in line with the current best practices in assisted reproduction, which are suggested by ESHRE guidelines (19).

Several limitations must be acknowledged. First, selection bias is a concern given the retrospective nature of the study. The 0PN-derived blastocysts analyzed were not representative of all the 0PN embryos initially observed, as they were early-stage embryos that survived the early stages of development, reached the blastocyst stage, and subsequently were selected for transfer or cryopreservation based on clinical and morphological criteria. As a result, this study should be regarded as describing the potential of a particular group of 0PN-derived blastocysts, not as describing a comparative analysis of all 0PN and all 2PN embryos. Second, time-lapse imaging was not available in the clinical setup, and therefore, pronuclear assessments were based on conventional static differential microscopy. Consequently, embryos classified as 0PN could have included early stages of pronuclear fading, asynchronous pronuclear appearance, and early stages of syngamy. As such, the 0PN group was biologically heterogeneous. Third, the absence of PGT-A represents an important limitation because the chromosomal status of 0PN-derived blastocysts could not be verified. 0PN-derived embryos may include biologically heterogeneous embryos resulting from early pronuclear fading, asynchronous pronuclear appearance, abnormal fertilization, haploidy, parthenogenetic activation, mosaicism, or aneuploidy. Therefore, we could not determine whether the transferred 0PN-derived blastocysts were chromosomally normal. This limits definitive interpretation of implantation, miscarriage-related outcomes, live birth, and long-term neonatal safety. Fourth. However, fragmentation, blastomere symmetry, and multinucleation were routinely assessed during embryo evaluation; complete quantitative and consistently retrievable records for these parameters were not available across the full retrospective cohort. This represents an important limitation because these morphology variables are established predictors of embryo competence and may confound or modify the association between Day-3 cell number and subsequent developmental potential. Therefore, the present findings should be interpreted as reflecting the prognostic value of Day-3 blastomere number/cleavage category rather than the independent effect of cleavage dynamics after full adjustment for all Day-3 morphology characteristics. The achieved power analysis further demonstrated that the available sample size did not reach 80% statistical power for detecting differences in live birth rate, and statistical power was particularly limited within Day-3 cleavage subgroups. This limitation is especially important for maternal and neonatal adverse outcomes, which were rare in the present cohort. Therefore, the absence of statistically significant differences should not be interpreted as evidence of equivalent safety or absence of risk. Instead, the findings should be regarded as preliminary and exploratory, requiring confirmation in larger, multicenter prospective studies with adequate power. Lastly, the absence of long-term neonate follow-up and clustering of patients may have impacted the embryo level outcomes of the study. Future prospective studies should include standardized and complete recording of Day-3 morphology variables, including fragmentation percentage, blastomere symmetry, and multinucleation, together with time-lapse monitoring, PGT-A where appropriate, and long-term offspring follow-up. Such studies would help determine whether Day-3 cell number remains independently associated with developmental and clinical outcomes after adjustment for other embryo morphology parameters.

## 5 Conclusion

The results here found that selected 0PN-derived blastocysts may be clinically relevant in terms of developmental and reproductive potentials, but completion rates for 0PN-derived blastocysts are still lower than those for 2PN-derived blastocysts. Day 3 embryo development dynamics provided further insight, and embryos reaching 7–9 cells showed the best potential for blastocyst formation and clinical outcomes in both 0PN and 2PN groups. Because of these results, cleavage-stage assessment should be used to direct storage and blastocyst stage decisions, but these results should not be used to justify the routine transfer of 0PN embryos. Although no statistically significant differences in short-term maternal or neonatal outcomes were detected between selected 0PN- and 2PN-derived blastocyst transfers, these findings should not be interpreted as evidence of equivalent safety. The limited number of 0PN live births, low incidence of adverse events, retrospective design, absence of routine genetic testing, and short neonatal follow-up substantially limit the ability to detect uncommon or long-term risks. In the absence of sui**Table 2**PN-derived blastocysts, selected 0PN-derived blastocysts may be considered cautiously in selected clinical circumstances; however, because of limited subgroup sample sizes and insufficient power to detect uncommon adverse outcomes, these findings should be interpreted as exploratory and require validation in larger, multicenter prospective studies incorporating standardized embryo selection, time-lapse monitoring, genetic testing, and long-term offspring follow-up.

## Data Availability

The datasets generated and/or analyzed during the current study are available from the corresponding author upon reasonable request.

## References

[1] Zhang X Chao S Ye N Ouyang D . Emerging trends in sperm selection: enhancing success rates in assisted reproduction. Reprod Biol Endocrinol. (2024) 22:67. doi: 10.1186/s12958-024-01239-1 38877490 PMC11177495

[2] Ghelardi C . Non-invasive biomarkers of embryo quality: How they can contribute to the success of Assisted Reproduction Techniques. (2022).

[3] Kemper JM Liu Y Afnan M Mol BW Morbeck DE . What happens to abnormally fertilized embryos? A scoping review. Reprod BioMedicine Online. (2023) 46:802–7. doi: 10.1016/j.rbmo.2023.02.005 36997399

[4] Chen C Li W Yin M Li M Wu L Si J . Does the cell number of 0PN embryos on day 3 affect pregnancy and neonatal outcomes following single blastocyst transfer? BMC Pregnancy Childbirth. (2022) 22:200. doi: 10.1186/s12884-022-04492-7 35279109 PMC8918324

[5] Ji H Bai Q Ding L Jiang L Shi Y Wang L . Prediction of blastocyst development using cleavage-stage embryo metrics and maternal age. Sci Rep. (2025) 15:24511. doi: 10.1038/s41598-025-10298-2 40629024 PMC12238512

[6] Yao G Xu J Xin Z Niu W Shi S Jin H . Developmental potential of clinically discarded human embryos and associated chromosomal analysis. Sci Rep. (2016) 6:23995. doi: 10.1038/srep23995 27045374 PMC4820740

[7] Bartolacci A Pavone V Barbagallo BM Cecchele A Papaleo E Pagliardini L . Reconsidering the clinical value of 1PN-derived embryos: a systematic review and meta-analysis. J Assisted Reprod Genet. (2025) 42:3619–32. doi: 10.1007/s10815-025-03631-1 40844726 PMC12640292

[8] Xu Q Mao X Zhang J Wu L . Nonpronuclear-and monopronuclear-derived blastocysts do not impair subsequent perinatal and maternal outcomes. J Clin Endocrinol Metab. (2025) 110:e953–61. doi: 10.1210/clinem/dgae375 38832947

[9] Guo H Sun X Jia Y Du J . Factors associated with the occurrence of 0PN fertilization in *in vitro* fertilization and its effects on embryo development and reproductive outcomes. Clin Exp Obstetrics Gynecology. (2024) 51:32. doi: 10.31083/j.ceog5102032

[10] Coticchio G Ahlström A Arroyo G Balaban B Campbell A De Los Santos MJ . The Istanbul consensus update: a revised ESHRE/ALPHA consensus on oocyte and embryo static and dynamic morphological assessment. Hum Reprod. (2025) 40:989–1035. doi: 10.1093/humrep/deaf021 40288770 PMC12127515

[11] Zhu M Dong Q Zhu Y Le Y Wang T Zhou Y . Developmental potential of non-and mono-pronuclear zygotes and associated clinical outcomes in IVF cycles. Front Endocrinol. (2024) 15:1361734. doi: 10.3389/fendo.2024.1361734 38532894 PMC10964251

[12] Moon JH Zhang J Yu B . Developmental potential of zygotes without pronucleus (0PN) and pregnancy outcomes after single euploid blastocyst transfers. J Assisted Reprod Genet. (2025) 42:1309–16. doi: 10.1007/s10815-025-03430-8 39984800 PMC12055727

[13] Destouni A Dimitriadou E Masset H Debrock S Melotte C Van Den Bogaert K . Genome-wide haplotyping embryos developing from 0PN and 1PN zygotes increases transferrable embryos in PGT-M. Hum Reprod. (2018) 33:2302–11. doi: 10.1093/humrep/dey325 30383227 PMC6238370

[14] Rao J Qiu F Tian S Cai Y Feng C Jin F . Clinical value of transferring blastocysts derived from 0PN/1PN or grade III embryos. Int J Clin Exp Med. (2020) 13:6945–52.

[15] Zhu Q Lin J Gao H Wang N Wang B Wang Y . The association between embryo quality, number of transferred embryos and live birth rate after vitrified cleavage-stage embryos and blastocyst transfer. Front Physiol. (2020) 11:930. doi: 10.3389/fphys.2020.00930 32922305 PMC7456822

[16] Si J Zhu X Lyu Q Kuang Y . Obstetrical and neonatal outcomes after transfer of cleavage-stage and blastocyst-stage embryos derived from monopronuclear zygotes: a retrospective cohort study. Fertility Sterility. (2019) 112:527–33. doi: 10.1016/j.fertnstert.2019.04.045 31272721

[17] Dar S Lazer T Shah P Librach C . Neonatal outcomes among singleton births after blastocyst versus cleavage stage embryo transfer: a systematic review and meta-analysis. Hum Reprod Update. (2014) 20:439–48. doi: 10.1093/humupd/dmu001 24480786

[18] Ji H Zhou Y Cao S Zhang J Ling X Zhao C . Effect of embryo developmental stage, morphological grading, and ploidy status on live birth rate in frozen cycles of single blastocyst transfer. Reprod Sci. (2021) 28:1079–91. doi: 10.1007/s43032-020-00381-6 33174188

[19] Xiong F Sun Q Wang S Yao Z Chen P Wan C . A nomogram to assist blastocyst selection in vitrified‐warmed embryo transfer cycles. J Obstetrics Gynaecology Res. (2022) 48:1816–28. doi: 10.1111/jog.15138 35373468

